# Uterine activity modifies the response of the fetal autonomic nervous system at preterm active labor

**DOI:** 10.3389/fendo.2022.1056679

**Published:** 2023-01-13

**Authors:** Rocio Lizbeth Olmos-Ramírez, Miguel Ángel Peña-Castillo, Hugo Mendieta-Zerón, José Javier Reyes-Lagos

**Affiliations:** ^1^ Basic Sciences and Engineering Division, Metropolitan Autonomous University (UAM) Campus Iztapalapa, Mexico City, Mexico; ^2^ Health Institute of the State of Mexico (ISEM), “Mónica Pretelini Sáenz” Maternal-Perinatal Hospital, Toluca, Mexico; ^3^ School of Medicine, Autonomous University of the State of Mexico (UAEMéx), Toluca, Mexico

**Keywords:** preterm labor, fetal heart rate variability, uterine contractions, continuous wavelet transform (CWT), autonomic function

## Abstract

**Background:**

The autonomic nervous system of preterm fetuses has a different level of maturity than term fetuses. Thus, their autonomic response to transient hypoxemia caused by uterine contractions in labor may differ. This study aims to compare the behavior of the fetal autonomic response to uterine contractions between preterm and term active labor using a novel time-frequency analysis of fetal heart rate variability (FHRV).

**Methods:**

We performed a case-control study using fetal R-R and uterine activity time series obtained by abdominal electrical recordings from 18 women in active preterm labor (32−36 weeks of gestation) and 19 in active term labor (39−40 weeks of gestation). We analyzed 20 minutes of the fetal R-R time series by applying a Continuous Wavelet Transform (CWT) to obtain frequency (HF, 0.2−1 Hz; LF, 0.05−0.2 Hz) and time-frequency (Flux0, Flux90, and Flux45) domain features. Time domain FHRV features (SDNN, RMSSD, meanNN) were also calculated. In addition, ultra-short FHRV analysis was performed by segmenting the fetal R-R time series according to episodes of the uterine contraction and quiescent periods.

**Results:**

No significant differences between preterm and term labor were found for FHRV features when calculated over 20 minutes. However, we found significant differences when segmenting between uterine contraction and quiescent periods. In the preterm group, the LF, Flux0, and Flux45 were higher during the average contraction episode compared with the average quiescent period (p<0.01), while in term fetuses, vagally mediated FHRV features (HF and RMSSD) were higher during the average contraction episode (p<0.05). The meanNN was lower during the strongest contraction in preterm fetuses compared to their consecutive quiescent period (p=0.008).

**Conclusion:**

The average autonomic response to contractions in preterm fetuses shows sympathetic predominance, while term fetuses respond through parasympathetic activity. Comparison between groups during the strongest contraction showed a diminished fetal autonomic response in the preterm group. Thus, separating contraction and quiescent periods during labor allows for identifying differences in the autonomic nervous system cardiac regulation between preterm and term fetuses.

## 1 Introduction

Preterm birth is defined as birth before 37 weeks of gestation (WG). It affects 5-18% of pregnancies worldwide and is the second cause of childhood death below five years (1). Preterm birth could be considered a pathological state that can cause health complications for life, most of them related to immaturity (2–5).

Since the fetal heart rate (FHR) is modulated by the autonomic nervous system (ANS), the fetal heart rate variability (FHRV) is an indirect way of measuring fetal autonomic activity (6). Analysis of FHRV can be done by spectral analysis that quantifies periodic changes in the FHR. This type of analysis estimates the power spectrum of representative bandwidths of the FHRV time series that determine the low-frequency (LF) or high-frequency (HF) components (7). In adults, the LF components are influenced by sympathetic and parasympathetic nervous system fluctuations, while the high-frequency (HF) components mainly reflect parasympathetic nervous system fluctuations (8).

Specifically, the spectral analysis of the FHRV has been successfully applied along pregnancy. Previous reports indicate that the FHRV power spectrum increase in both HF and LF throughout pregnancy (non-labor), which has been associated with fetal autonomic development (9, 10). Along the same line, a study used power spectrum analysis of FHRV in post-term (≥42 WG) and near-term (36-37 WG) fetuses during the first stage of labor. The authors found that near-term fetuses showed a sympathetic predominance during the active state, while post-term fetuses showed higher parasympathetic modulation during the quiet state. The study concluded that a higher resting parasympathetic activity is manifested in post-term fetuses than in near-term fetuses, probably associated with higher autonomic neurodevelopment in the post-term fetuses (11). Thus, the FHRV power spectrum analysis has also shown the potential to assess fetal surveillance, especially to detect high-risk conditions such as fetal acidemia (12, 13). Additional findings reported that the FHRV power spectrum of acidotic preterm fetuses behaves differently during labor than that of acidotic term fetuses (14).

In general, it can be said that, as described in adults, the fetal heart rate can change in a particular manner in the presence of specific external stimuli (15, 16), which makes it useful to evaluate the maturation of the fetal autonomous nervous system or ANS in the presence or absence of uterine activity. During labor, uterine contractions correspond to unavoidable stimuli, increasing the FHRV power spectrum (17). The fetal response to uterine contractions is related to the fetal capability against a temporarily reduced oxygen supply due to umbilical cord occlusion or reduced maternal placental blood flow (18). The contractions also cause significant fluctuations in the intrauterine pressure, directly influencing the fetal cardiovascular system (19). Intermittent hypoxemia during labor causes a repetitive activation of the peripheral chemoreflex (20). Under normal conditions, fetuses can overcome the stressful process of labor through homeostatic processes mediated by neuroendocrine mechanisms. However, the failure of these protective mechanisms could cause critical damage to the fetuses (21). We consider that specific attention should be taken to the particular response to contractions in preterm fetuses that may have a less developed response of such protective mechanisms (22).

A way to assess the preterm fetal-specific response to uterine contractions is by utilizing FHRV analysis. Nevertheless, the FHRV time series during active labor is highly nonstationary (23). Unlike studies in adults where the same controlled conditions are attempted between individuals, in the study of FHRV during labor it is impossible to control the timing of external stimuli, such as fetal movements or uterine contractions. However, despite these complications, a relevant study found that separating the contraction and non-contraction periods can help to analyze the response to labor and to improve the detection of fetal distress (24, 25). A suitable method for processing high nonstationary signals is the time-frequency analysis. This analysis helps to study frequency components along the time series and can be addressed by a mathematical tool named Continuous Wavelet Transform (CWT) (26). We propose that the spectral analysis of the FHRV can also be performed by extracting the frequency bands from the time-frequency representation by applying the Inverse Continuous Wavelet Transform (ICWT). This method allows calculating the power of different frequency components of the original R-R time series while conserving the time resolution (27).

Our previous study showed a different autonomic response in preterm than term fetuses during active labor using asymmetric FHRV analysis methods. Thus, preterm fetuses seem to experience more decaying R-R trends and a lower magnitude of decelerations than term fetuses. These differences in FHR dynamics could be related to the immaturity of the fetal autonomic nervous system as identified by this system’s response to the intense uterine activity at active labor (28). It has been described that separating uterine contraction and quiescent periods could be helpful to discriminate fetuses in distress (29). In this work, we analyze if the preterm fetal response to contraction describes a level of immaturity because currently, there is a pressing need to create non-invasive techniques that may be employed intrapartum during the term and preterm periods offering complementary information on fetal health.

According to our literature review, the specific autonomic response of preterm fetuses to uterine contractions during labor has not been addressed. This study aims to compare the behavior of the fetal autonomic response to uterine contractions between preterm and term active labor using a novel time-frequency analysis of fetal heart rate variability. As a secondary objective, our study compares linear, nonlinear, and time-frequency indices of FHRV by discriminating high uterine contraction and quiescent periods in preterm and term fetuses, respectively. We hypothesized that CWT enables the analysis of the cardiac autonomic response of term and preterm fetuses in active labor because it has been of help for the study of nonstationary physiological time series. We also consider that the fetal autonomic response caused by uterine contractions in labor may differ between term and preterm fetuses owing to the fact that the usual health complications at birth from the latter could well be related to a lower ANS modulation.

## 2 Materials and methods

### 2.1 Dataset description and preprocessing

The dataset from this study was obtained at the “Monica Pretelini Sáenz” Maternal-Perinatal Hospital in Toluca de Lerdo, Mexico State, Mexico, over the period 2018-2020, as described in our previous study (28). The ethics committee from this institution approved this study (reference number: 2018-10-607). Written informed consent was obtained from each participant, and the study was conducted according to the ethical standards of the Declaration of Helsinki and following relevant guidelines and regulations. All registrations (n=78) were recorded with a fetal-maternal monitor (Monica AN24, Monica Healthcare, Nottingham, UK) during active labor in singleton pregnancies. We considered active labor once manifesting at least four uterine contractions in 10 minutes, 4 cm dilatation, and 50% cervical effacement. Mothers with a history of preeclampsia, heart disease, smokers, and drug abuse were excluded from the study. Administration of oxytocin or anesthesia was also an exclusion criterion.

We extracted the R-R interbeat interval and uterine electrical activity (TOCO-like) signals from recorded transabdominal data of pregnant women during active labor at term (37–40 WG) and women at preterm labor (32–36 WG). The length of the recordings was between 10 and 60 minutes. Nevertheless, given the difficulties of finding long recordings of transabdominal ECG without missing data, we decided to select the 20 minutes with the smallest loss of data for each recording. Signal extraction was done using the Monica DK software (Monica Healthcare, Nottingham, UK). The fetal R-R interbeat interval signals with low quality were excluded from the analysis, including recordings with a high missing rate (> 10% of missed fetal beats) and recordings with less than 20 min. We selected for the analysis the recordings of fetuses up to 36 weeks of gestation (32-36 WG) for the preterm group (PT group) and those at least 39 weeks of gestation (39-40 WG) for the term group (T group).

Ectopic beats were eliminated by adaptive filtering (30). This algorithm consisted of two sequential filters: An adaptive percentage filter based on the adaptive mean and standard deviation and an adaptive control filter to correct points in the series that differ by more than three standard deviations from the adaptive mean value. We detrended the fetal R-R interval time series to remove the DC component. Subsequently, a cubic spline interpolation at 4 Hz was applied to obtain uniformly sampled fetal R-R time series and equidistant TOCO-like signals. Next, we used the CWT to obtain the time-frequency representation of a 20-minute segment of the R-R interval signal using the analytic Morse wavelet as a mother function (27) as implemented in the MATLAB version R2020b (The MathWorks Inc, Natick, MA, USA).

The CWT generates a time-frequency representation by analyzing the time series at different frequencies with different time resolutions. The analysis of a time series by the CWT requires a set of functions generated from a base function *ψ* (t) or mother wavelet. A family of functions *ψ*_*a*,*b*_ (t) is obtained by dilatation and translocation, varying its scale (a) and translation (b) parameters as follows (26):


(1) $$\psi_{a,b}\left(t\right)=\frac{1}{\sqrt{a}}\psi \left(\frac{t-b}{a}\right)$$

where *a* is a real number different from zero, *b* is a real number, and *t* is the time value.

Then the CWT of a signal *s(t)* is defined as the convolution of *s(t)* with the complex conjugate of the wavelet family *ψ*_a,b_(t), as is expressed as follows:


(2) $$S\left(b.a\right)=\frac{1}{\sqrt{a}}\int_{-\infty }^{\infty }\psi^{\prime }\left(\frac{t-b}{a}\right)s\left(t\right)dt$$

The time-frequency representation obtained by CWT was constituted with a minimum frequency of 0.001 Hz and a maximum frequency of 1.47 Hz.

From the time-frequency representation, we calculated the frequency and time-frequency domain features. All features of FHRV were estimated over 20 minutes segments (short-term analysis) and from all contraction and quiescent periods occurring in 50 s windows (ultra-short-term analysis). We averaged the features obtained from each recording contraction and quiescent periods to study the average fetal response to contractions. We also analyzed the contraction periods in which the strongest contraction of each recording was manifested once, considering that it represents the fetal response to the highest stimulus (a single 50-second segment per recording).

**Figure 1:**
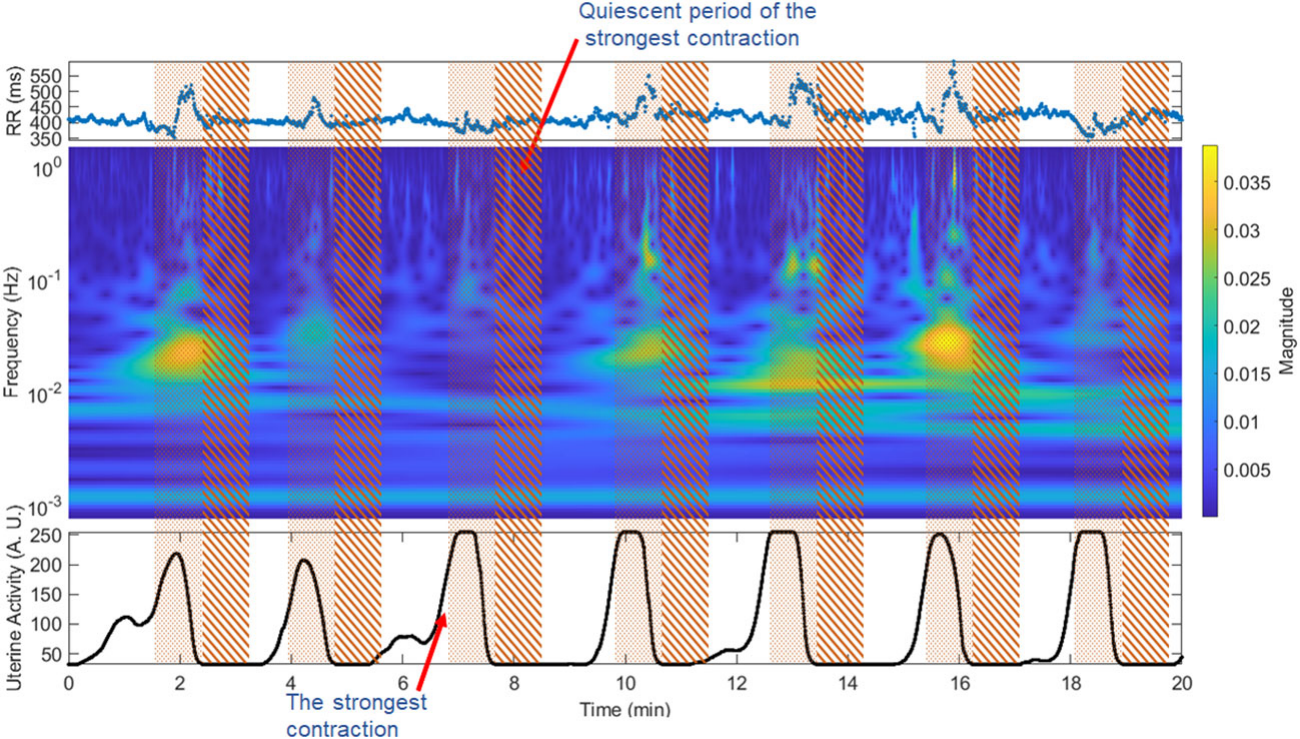
Window segmentation of 50 seconds for the fetal R-R signal and time-frequency representation. Pointed area: contraction period, striped area: quiescent interval. The upper panel shows the fetal beat-to-beat R-R time series; the middle panel shows the time-frequency representation of the fetal R-R time series; the lower panel shows the TOCO-like signal.

We used the TOCO-like signal to define the two relevant uterine activity periods: the presence of high electrical uterine activity (contraction) and the absence of uterine electrical activity (quiescent). We first delimited the uterine contractions in the intervals where the uterine activity signal increased above 32 arbitrary units (AU), whose maximum value was superior to 50 AU (contractions) according to previous studies (31). To avoid variation caused by different durations of contraction or quiescent periods, we used 50 s windows that cover more than two times the period’s length of the lowest frequency of interest (0.05 Hz). The R-R intervals were segmented 50 seconds before the endpoint of the contraction period (contraction period) and 50 seconds after the end of contractions (quiescent period). We thus calculated one quiescent period for each contraction (**Figure 1**). In addition, given that the time-frequency representation keeps the time relation, we delimited the study segments directly on the time-frequency representation to calculate the corresponding features.

The strongest contraction was selected by comparing the maximum value of the contraction periods and designating the period with the highest amplitude of the TOCO-like signal (**Figure 1**). Given that the maximum value displayed by the Monica DK software is 255 AU, in some cases the recordings presented more than one period with this maximum number. Hence, we selected only the first of them.

### 2.2 Time-frequency domain features

Time-frequency representations can be treated as an image *p[n, m]* with dimensions *N×M*, where *N* and *M* are the maximum frequency and time sampling points, respectively. We calculated the features of Flux and Renyi Entropy (32).

The Flux describes the accumulated rate of change. It was defined in three directions as Flux 0, Flux 45, and Flux 90, which represent the trend of change over time, time-frequency, and frequency, respectively, as follows:


(3) $$Flux0=\sum_{n=1}^{N}\sum_{m=1}^{M-1}\left|p_{nm+1}\left|-\right|p_{nm}\right|$$

(4) $$Flux45=\sum_{n=1}^{N-1}\sum_{m=1}^{M-1}\left|p_{n+1m+1}\left|-\right|p_{nm}\right|$$

(5) $$Flux90=\sum_{n=1}^{N-1}\sum_{m=1}^{M}\left|p_{n+1m}\left|-\right|p_{nm}\right|$$

Normalized Renyi entropy measures the randomness of the power:


(6) $$Entropy_{R}=-\frac{1}{2}log\left[\sum_{n}\sum_{m}{\left(\frac{p_{nm}}{\sum_{n}\sum_{m}\left(p_{nm}\right)}\right)}^{3}\right]$$

### 2.3 Frequency−domain features

**Figure 2:**
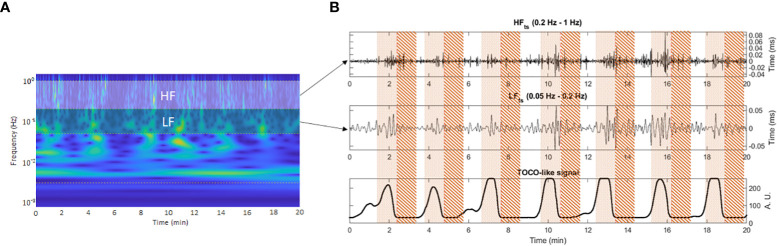
Window segmentation of 50 seconds on the representative frequency bands time series. Pointed area: contraction interval of study. Striped area: quiescent interval of study. Panel **(A)** shows the frequency band delimitation on the time-frequency representation of the R-R time series. Panel **(B)** shows contraction and quiescent analysis window delimitation over the frequency bands time series (upper and middle subplots) aligned with the TOCO-like signal (lower subplot).

We calculated the frequency−domain features from the R-R time series corresponding to representative frequency bands extracted by applying the CWT and the ICWT sequentially to extract the LF (0.05−0.2 Hz) and HF (0.2−1 Hz) bands time-frequency representation (27). The wavelet coefficients in the time-frequency representation were zeroed below and above each bandwidth of interest. The resulting nonzero coefficients were transformed back to the time domain by applying the ICWT. Subsequently, we obtained the high and low-frequency bands time series (HF_ts_ and LF_ts_), which were used to calculate HF and LF indices over the analyzed segments (**Figure 2**). The spectral indices of HF and LF were computed as the root mean squared (RMS) of their respective time series.

### 2.4 Time-domain features

Time-domain indices were calculated directly from the R-R time series after removing ectopic beats by adaptive filtering (30). The mean value (meanNN), the standard deviation (SDNN), and the root mean square of the differences between successive samples (RMSSD) were calculated with a MATLAB implementation (33). The meanNN measures the mean value of fetal heart rate; SDNN measures global heart rate variability and reflects sympathetic and parasympathetic regulation. While RMSSD primarily reflects parasympathetic regulation, describing short-term variations in the time domain (34).

### 2.5 Statistical analysis

The Shapiro-Wilk test was applied to test the data normality. Clinical characteristics were statistically compared (p<0.05) using a Chi-square test for nominal variables (APGAR and biological sex). A t-test for continuous variables with normal distribution was applied (head circumference, BMI, number, and intensity of contractions), and the Wilcoxon rank-sum test was applied to not normal continuous variables (dilation, effacement, height, and weight). Since feature values were not normally distributed, the statistical analyses were performed with the Wilcoxon-signed ranks test to verify significant differences between the distributions of FHRV indices during quiescent and contraction periods. We also used the Mann-Whitney U test to determine significant differences between the term and preterm groups. Statistical significance was set at a p-value of less than 0.01.

## 3 Results

### 3.1 Clinical characteristics

From the total transabdominal records in the dataset (n=78), we only selected 18 cases from the preterm labor (PT) group, and 19 from the term labor (T) group since some of the records were not suitable for analysis. Either because they were shorter than 20 minutes (8 cases), had more than 10% of lost data (18 cases), or their gestational age was between 37 and 39 WG (15 cases). The clinical characteristics of the study population are summarized in **Table 1** as mean, standard deviation and interquartile range. Significant differences are shown as *. Newborns’ measurements of length (p=0.018), weight (p= 0.003), and gestational age (p<0.0001) were significantly lower in the PT group in comparison with the T group. No significant differences between the T and PT groups in maternal clinical characteristics were found, indicating that both groups are similar. In addition, the values of cervical dilatation and the number of contractions in 20 minutes (between 5 and 10) confirmed that data was collected in active labor for both the PT and T groups.

**Table 1:** Clinical characteristics of newborns and mothers for Term (T) and Preterm (PT) groups (mean +/-SD), and interquartile range.

	PT (n=18)	T (n=19)
Newborn		
Gestational age (Weeks) *	34.58 ± 1.68, [33.7 36.15]	39.79 ± 0.75, [39.1 40.35]
Biological sex (male or female)	52.9% Male 47.1% Female	58.8% Male 41.2% Female
Length (cm) *	46.17 ± 5.63, [47 49]	49.7 ± 2.02, [49 51]
Head circumference (cm)	32.5 ± 2.04, [31.75 33.25]	33.70 ± 1.78, [33 35]
Weight (kg) *	2.53 ± 0.48, [2.41 2.76]	3.03 ± 0.4, [3.80 3.31]
APGAR 1 minute (points)	8 [8 9]	8 [8 8]
APGAR 5 minutes (points)	8 [7.75 9]	9 [8.25 9]
Mother		
Age (years)	24.33 ± 5.64, [19.25 27]	23 ± 5.99, [19 27]
BMI (kg/m^2^)	26.01 ± 3.51, [23.12 27.68]	25.18 ± 3.56, [22.9 25.45]
Cervical dilatation at labor (cm)	5.43 ± 1.45, [4.75 6.25]	5.36 ± 1.46, [4 6.5]
Cervical effacement at labor (%)	66.25 ± 13.10, [0.5 0.725]	73.15 ± 10.56, [0.6 0.8]
Number of contractions over 20 minutes	7.22 ± 1.89, [6 8]	7 ± 2.05, [5.25 8]
Contraction intensity (AU)	127.98 ± 60.16, [75.48 193.98]	126.08 ± 63.34, [80.24 174.48]

Values are expressed as mean, standard deviation, and in brackets the interquartile range.

*Represents statistical differences between PT and T groups (p<0.05).

### 3.2 Comparison over 20 minutes of recording (short-term analysis of FHRV)

No significant differences were found in any of the calculated indices in the statistical comparison between the two groups (T and PT) after processing the whole 20 minutes recording of the fetal beat-to-beat R-R time series (data shown in **Table 2** as median and interquartile range).

**Table 2:** Values of linear, nonlinear, and time-frequency indices of short-term fetal heart rate variability (FHRV) calculated over 20 minutes segments for Term (T) and Preterm (PT) groups.

	PT (N=18)	T (N=19)
RMSSD (ms)	7.73 [6.37 8.50]	6.84 [5.85 8.20]
SDNN (ms)	20.33 [17.28 27.27]	17.69 [16.1 19.96]
meanNN (ms)	412.17 [381.33 425.28]	418.97 [407.87 442.19]
HF (ms^2^)	6.39 [5.64 7.32]	5.91 [4.84 7.14]
LF (ms^2^)	10.50 [8.57 11.67]	8.80 [7.62 10.74]
Flux0	-19.85 [-44.57 148.08]	52.17 [-123.11 222.69]
Flux45	146.58 [-2237.48 1778.48]	800.93 [-1459.26 2229.90]
Flux90	74.48 [-2236.58 1741.91]	661.98 [-1562.71 2145.49]
Entropy_R_	14.61 [14.49 14.71]	14.49 [14.39 14.59]

Values are expressed as median and interquartile range [Q1 Q3] p>0.05.

### 3.3 Comparison within groups (contraction-quiescent, ultra-short-term analysis of FHRV)

When comparing the average values of the FHRV indices during contractions with the average values on their immediate subsequent quiescent periods, it was found that in both groups, the Entropy_R_ and SDNN (p=0.0009, p=0.007), respectively are significantly higher (p<0.01) during contraction periods (**Table 3**). In addition, in the PT group, it was found that LF, Flux0, and Flux45 were also significantly (p<0.01) higher during contractions. While in the T group, RMSSD (p=0.01) and HF (p=0.005) were higher in the contraction periods. Summarized data is shown in **Table 3** as median and interquartile range.

**Table 3:** Values of linear, nonlinear, and time-frequency indices of ultra-short-term fetal heart rate variability (FHRV) during average contraction and quiescent periods for Term (T) and Preterm (PT) groups.

	PT (N=18)		T (N=19)	
	Contraction	Quiescent	Contraction	Quiescent
SDNN (ms)	9.50 [7.95 12.59]*	7.55 [5.49 10.64]*	8.71 [7.25 12.92]*	6.63 [5.84 9.52]*
RMSSD (ms)	2.98 [2.33 3.82]	2.88 [2.00 3.27]	2.44 [1.84 3.65]*	2.24 [1.77 2.74]*
meanNN (ms)	257.30 [216.01 293.63]	253.13 [203.48 283.32]	266.80 [210.69 328.81]	266 [213.02 324.18]
HF (ms^2^)	3.91 [3.06 5.25]	3.59 [2.63 4.66]	3.38 [2.62 5.51]*	3.02 [2.09 4.06]*
LF (ms^2^)	5.77 [4.58 8.41]*	5.33 [3.33 6.66]*	5.40 [3.82 8.07]	4.27 [2.92 7.32]
Flux0	30.68 [5.51 63.99]*	-49.43 [-73.03 -12.43]*	6.46 [-15.14 25.17]	-17.63 [-52.70 10.76]
Flux45	9.75 [-58.27 81.28] *	-58.63 [-93.03 27.89] *	31.54 [-5.46 110.64]	-8.48 [-30.14 111.97]
Flux90	-9.39 [-65.90 27.02]	-7.21 [-63.60 44.75]	14.66 [-39.97 93.40]	12.09 [-28.12 101.89]
Entropy_R_	6.26 [5.42 7.14] *	5.87 [4.96 6.85] *	6.06 [4.89 7.22] *	5.32 [4.90 7.14] *

Values are expressed as median and interquartile range [Q1 Q3].

*Represents differences within PT and T groups (contraction vs. quiescent, p<0.01).

Significant differences were also found while comparing each record’s strongest contraction and the corresponding immediate consecutive quiescent period (One 50s segment per record). In the term group, the difference between contraction and quiescent periods was significantly higher for Entropy_R_, SDNN, RMSSD, HF, and LF in the contraction periods. While in the preterm group, meanNN was the only feature that showed significant differences between contraction and their immediate posterior quiescent periods. Summarized data is shown in **Table 4** as median and interquartile range.

**Table 4:** Values of linear, nonlinear, and time-frequency indices of ultra-short-term fetal heart rate variability (FHRV) during the strongest contraction and the consecutive quiescent period for Term (T) and Preterm (PT) groups.

	PT (N=18)		T (N=19)	
	Contraction	Quiescent	Contraction	Quiescent
SDNN (ms)	12.67 [10.57 20.53]	12.96 [8.89 18.27]	17.16 [14.68 27.90]*	10.03 [6.72 17.45]*
RMSSD (ms)	4.66 [3.19 5.74]	4.31 [2.98 6.13]	5.08 [3.28 6.45]*	3.73 [2.73 4.78]*
meanNN (ms) ♣♦	396.45 [379.29 413.81]*	401.02 [388.13 421.99]*	431.61 [404.34 456.31]	433.53 [404.83 453.46]
HF (ms^2^)	6.11 [3.48 7.94]	5.08 [3.63 6.88]	7.79 [4.64 10.03]*	4.98 [3.19 6.15]*
LF (ms^2^)	7.87 [6.02 10.61]	8.49 [5.89 10.39]	11.11 [7.83 15.03]*	6.76 [4.01 12.10]*
Flux0	18.40 [-44.77 102.51]	-67.23 [-162.69 40.50]	-18.20 [-102.82 46.14]	-4.87 [-134.94 35.48]
Flux45	-8.66 [-135.85 145.15]	-66.71 [-253.58 121.71]	39.85 [-154.62 154.62]	13.87 [-111.93 245.33]
Flux90	-33.96 [-102.63 59.81]	-40.95 [-90.94 80.20]	31.24 [-48.56 96.50]	18.41 [-44.63 173.87]
Entropy_R_	9.75 [9.60 10.17]	9.76 [9.45 10.07]	9.96 [9.77 10.12] *	9.61 [9.43 10.16] *

Values are expressed as median and interquartile range [Q1 Q3].

*Represents differences within PT and T groups (contraction vs. quiescent, p<0.01).

♦Represents differences between PT and T groups (quiescent vs. quiescent, p<0.01).

♣Represents differences between PT and T groups (contraction vs. contraction, p<0.01).

### 3.4 Comparison between groups (PT-T, ultra-short-term analysis of FHRV)

No significant differences were found in average indices between PT and T groups. However, in the analysis of the strongest contractions and their consecutive quiescent period, the meanNN was lower in the PT group during both contraction (p=0.0005) and quiescent (p= 0.0018) periods compared with the T group. Values are shown in **Table 4** as median and interquartile ranges.

## 4 Discussion

This study aimed to compare linear, nonlinear, and time-frequency features of heart rate variability to analyze the cardiac autonomic changes of preterm and term fetuses in response to a naturally stressful process such as uterine contractions during active labor. Previous reports indicated changes in the FHRV features during contractions in fetuses at term. Specifically, the power spectrum seems to increase by the contribution of all frequency bands in the presence of uterine contractions. Thus, authors have considered that calculating FHRV features of the R-R time series without considering the uterine activity may average the fetal response to contractions with the fetal activity during quiescent periods (17). Given that contractions influence the fetal cardiovascular system, the specific fetal adaptability to external stimuli can help to identify fetal distress, such as that caused by asphyxia. In these conditions, preterm fetuses could not adapt properly to stressful conditions as it occurs with low-risk fetuses (25, 29).

No differences were found in the fetal autonomic dynamics of PT and T groups over the span of 20 minutes. However, window segmentation of fetal R-R time series based on uterine activity helped to identify significant differences between the fetal autonomic response of term and preterm fetuses. Notably, the CWT also allowed us to identify changes in some spectral indices of FHRV. In the present study, our main findings showed that preterm fetuses exhibit different cardiac dynamics in response to uterine contractions than that presented by term fetuses. Preterm FHRV seems to change in the low-frequency components as represented by a higher LF, Flux0, and Flux45 during uterine contraction periods when the average values of the contractions are compared with their consecutive quiescent periods. Otherwise, in the T group, changes are mainly observed in the representative vagally mediated features (HF and RMSSD). These findings suggest that the parasympathetic branch could mediate the autonomic control of the fetal heart rate in term fetuses, and the sympathetic/parasympathetic branches could dominate the autonomic control in preterm fetuses.

Lear et al., suggested that the fetal cardiac response to frequent contractions is mediated by the parasympathetic branch once that is considered that the sympathetic branch is temporally suppressed by negative feedback pathways (35). Our results showed that this could be indeed the case for the term group. Meanwhile, the differences in response in the preterm group could be related to the immaturity of the parasympathetic branch. Relevant findings indicate that preterm fetuses may have a higher baseline fetal heart rate and an apparent reduction in baseline variability because of the unopposed action of the sympathetic nervous system (34). Following those results, further evidence also suggests that preterm newborns exhibit lower parasympathetic modulation of the heart (35). According to Patural et al., high-frequency fetal dominance continues to increase until the last months of pregnancy, and preterm newborns manifest lower parasympathetic activity than full-term newborns (36), which may have a decreased sympathetic influence owing to an increase in the vagal influence (37).

It has been described that a rapid increase in vagal activity occurs around 37 to 38 WG, which is appreciated as an increased high-frequency variability related to the development of respiratory sinus arrhythmia (RSA) caused by fetal respiratory-like movements (36). Although this study was conducted during labor, where fetal respiratory movements are diminished, our findings in HF could rather be related to the contraction stimuli and not produced by fetal respiratory movements.

Flux0 and Flux45 indices reflect the trends of the instantaneous power change over both frequency and time, which could be associated with the number of HR-positive accelerations (accelerations) and negative accelerations (decelerations) in those periods. The difference observed in the dynamics of Flux0 ​​between the PT and T groups for the average values during contractions and their quiescent periods indicates a greater unidirectional change within the PT group’s response to contractions. The positive average value in Flux0 during contractions in PT indicates a positive trend in energy changes of the R-R series that would be identified as a deceleration. It is crucial to notice that the opposite tendency compensates for such behavior by restoring HR values during immediate quiescent periods. This behavior is similar to what has been described as the heart compensation to acute hypoxia, where the fetal heart rate temporarily diminishes to reduce fetal oxygen consumption (37). A specific directionality during a relatively large time scale of 50 s is related to the predominance of low-frequency content.

On the other hand, term fetuses did not show a specific trend of change in Flux0, indicating higher variability in the instant energy changes over time that could be related to higher autonomic maturity (36). Even though the Flux45 sign does not reflect a specific trend given the dispersion of its values, the differences found between contraction and quiescent periods in the preterm group show a change in frequency predominance over time, an indicator of a different specific response within this group, which could be of interest. Although no difference was found while statistically comparing the average values of both groups, the specific analysis within groups showed important distinctions in fetal response within our study groups.

In the analysis of fetal response to the strongest contraction compared with its consecutive quiescent period, we found that the T group manifested a higher Entropy_R_, SDNN, RMSSD, HF, and LF, during the contraction period indicating transient changes in the autonomic activity of both ANS branches as a response to the highest stimulus. These results could be related to previous descriptions of increased vagal modulation when sympathetic activation is present in term fetuses (36). Comparatively, the PT group did not show the same response; only a lower meanNN during the strongest contraction was observed compared to the consecutive quiescent period. The changes in the meanNN could be related to a robust adrenergic response that the immature vagal tone cannot modulate. The differences in the meanNN index when comparing both contraction and quiescent periods between T and PT groups further support this hypothesis. Comparatively, during the strongest contraction, the preterm group seems to behave similarly to parasympathetic blocked subjects in preclinical studies. For example, parasympathetic blocked sheep fetuses (through vagotomy or atropine administration) present higher FHR during and after umbilical cord occlusions in comparison with a control group (non-blocked fetuses) (35, 38).

The analysis of the most vigorous contractions showed a different behavior in these contractions than the average values, which may be related to a sympathetic suppression after transient hypoxemia described in Lear et al., study (35). However, this should be taken carefully, given that the changed FHRV behavior through different contractions could also be related to a different amount of transient hypoxia caused by each contraction, which we did not measure. The reduction of meanNN shows a deceleration in the R-R signal that was not identified by the Flux0 index, which means that it was a deceleration faster than 50 s. Specific reactions like this could be why preterm fetuses show greater manifestation of decelerations than term fetuses while analyzing the proportions of accelerations and decelerations in continuous R-R segments (28). In these cases, the reduction in the meanNN continues even after the end of the strong contraction stimulus. Even though no strong evidence exists that continuous strong contractions could affect newborns’ outcomes, specifically at term (39), our results suggest that specific attention should be taken to strong and repetitive contractions in preterm labor, given that their response seems to have less autonomic modulation concerning term fetuses. Although further investigations are needed to address specific implications of continuous strong contractions in preterm fetuses outcomes.

## 5 Conclusion

Our results indicate that the average autonomic response to contractions in preterm fetuses shows a sympathetic predominance, while term fetuses rather respond through a parasympathetic activity. However, specific reactions to the most vigorous contractions were found while comparing the preterm and term groups showing a diminished fetal autonomic response in the preterm group, which could be associated with a reduced capacity to adapt to this strong stimulus that could lead to health complications in the newborn. This work demonstrates that separating contraction and quiescent periods during labor allows to identify differences in the autonomic nervous system cardiac regulation between preterm and term fetuses that have not been visualized by studying more extended periods with linear indices of fetal heart rate variability.

## Data availability statement

The raw data supporting the conclusions of this article will be made available by the authors, without undue reservation.

## Ethics statement

The studies involving human participants were reviewed and approved by the ethics committee from the “Monica Pretelini Sáenz” Maternal-Perinatal Hospital in Toluca de Lerdo, Mexico State, Mexico (reference number: 2018-10-607). The patients/participants provided their written informed consent to participate in this study.

## Author contributions

JR-L and MP-C, conceived the presented idea. RO-R, developed the theory and performed all the computations. JR-L, MP-C, and HM-Z verified the analytical methods. All authors contributed to the article and approved the submitted version.
